# Sex differences in the association of vascular risk and APOE Genotype with cognitive decline and dementia: evidence from a U.S. longitudinal study

**DOI:** 10.1016/j.lana.2025.101346

**Published:** 2025-12-26

**Authors:** Longjian Liu, Jintong Hou, Saishi Cui, Xiaopeng Zhao, Zuolu Liu, J. Craig Longenecker, Nathalie S. May, Jin Jun Luo, Rose Ann DiMaria-Ghalili, Howard J. Eisen

**Affiliations:** a Department of Epidemiology and Biostatistics, Dornsife School of Public Health, Drexel University, Philadelphia, PA, 19104, USA; b Department of Mechanical, Aerospace, and Biomedical Engineering, University of Tennessee Knoxville TN, 37996, USA; c Neurology, California Pacific Medical Center, Sutter Health, San Francisco, CA, 94109, USA; d Department of Medicine, College of Medicine, Drexel University, Philadelphia, PA, 19104, USA; e Department of Neurology, Lewis Katz School of Medicine at Temple University, Philadelphia, PA, 19140, USA; f Doctoral Nursing Department, College of Nursing and Health Professions, Drexel University, Philadelphia, PA, 19104, USA; g Clinical Research for the Advanced Cardiac and Pulmonary Vascular Disease Program, Thomas Jefferson University Hospital, Philadelphia, PA, 19107, USA

**Keywords:** Sex differences, Vascular risk, Genetic risk, Cognitive decline, Dementia, Longitudinal study

## Abstract

**Background:**

Sex differences in the association between vascular factors and cognitive outcomes remain unclear. We aimed to investigate the associations of blood pressure metrics (hypertension, systolic blood pressure [SBP), pulse pressure, ankle and brachial pressures, and ankle to brachial pressure index [ABI]) with the risk of cognitive decline and dementia.

**Methods:**

We conducted a population-based longitudinal analysis using data from the Atherosclerosis Risk in Communities (ARIC) study (begun in 1987–1989) in the United States. We analyzed a total of 12,268 participants aged 45–64 years who had validated exposure measurements, cognitive function tests (first administrated 1990–1992), and followed up for incidence of dementia through December 2019. Cognitive function was assessed using the Digit Symbol Substitution Test, the Delayed Word Recall Test, and the Word Fluency Test. Dementia cases were identified through a standardized clinical evaluation process, mostly adjudicated by expert reviewers. We performed sex-stratified analyses to examine the associations of blood pressure metrics and APOE ε4 allele with the risk of cognitive decline and dementia.

**Findings:**

Over a median follow-up of 26.4 years, 2698 participants developed dementia. Women aged 55–64 had a significantly higher incidence of dementia than men aged 55–64 (14.8 vs. 11.8 per 1000 person-years; p < *0.0001*). After adjusting for key covariates, SBP, pulse pressure, ankle and brachial pressures were significantly associated with cognitive decline in women (p < 0.05). In men, pulse pressure and ankle pressure showed a significant association. In longitudinal analyses, baseline hypertension and elevated brachial pressure were significantly associated with increased dementia risk in women, whereas in men, elevated brachial pressure and decreased ABI were significantly associated with dementia risk. Individuals with APOE ε4 allele had significantly higher risk of dementia in both sexes. A joint effect of APOE ε4 allele and elevated blood pressure metrics conferred a greater relative excess risk of dementia in women vs. men.

**Interpretation:**

These findings highlight notable sex differences in the association between vascular factors and cognitive decline and dementia risk. Women appear more vulnerable to both genetic and vascular risk factors, emphasizing the need for sex-specific approaches in research, prevention, and intervention strategies for cognitive impairment.

**Funding:**

10.13039/100000002NIH.

**Research in context**

**Evidence before this study**

Previous community-based prospective studies investigating the associations between vascular factors and cognitive decline and dementia have reported inconsistent findings. We searched PubMed on May 16, 2025, using the terms (“sex differences AND vascular factors AND cognitive decline OR dementia” OR “sex differences AND blood pressure AND cognitive decline OR dementia” OR “sex differences AND cardiometabolic disorder AND cognitive decline OR dementia”) for English-language studies that examined the sex-specific effects of vascular factors on cognitive decline and dementia risk. The results revealed contradictory evidence.

**Added value of this study**

Using data from one of the largest community-based cohort studies in the United States, we provide robust evidence that the combined impact of vascular and genetic risk factors, specifically the presence of the APOE ε4 allele, yields a significantly stronger association with cognitive decline and dementia risk in women than in men.

**Implications of all the available evidence**

Our findings underscore the importance of sex-specific approaches in assessing and managing vascular risk factor to prevent cognitive decline and dementia. The stronger associations observed in women, especially *APOE* ε4 carriers, suggest that targeted screening for blood pressure metrics in midlife may offer a critical window for monitoring association with changes in cognitive function and for intervention against dementia. Clinicians should recognize that even subclinical vascular changes can lead to long-term neurocognitive consequences, with disproportionate effects in women. From a policy perspective, these results support the development of tailored public health strategies that incorporate biological sex, genetic risk, and early measures of blood pressure metrics into dementia prevention programs. Equitable access to vascular health screening, improving education on modifiable risk factors, and ensuring culturally appropriate care are essential. Incorporating sex-based risk stratification in clinical guidelines may enhance the effectiveness of early prevention strategies and reduce disparities in dementia outcomes.

## Introduction

The global burden of cognitive decline and dementia is rapidly increasing with aging populations, posing a major public health challenge.^1^ In the United States alone, the prevalence of Alzheimer's disease, the most common form of dementia, is projected to more than double over the coming decades.^2^ Cognitive decline often precedes clinical dementia, offering a critical window for early detection and intervention. Therefore, identifying modifiable risk factors for cognitive decline and dementia is essential to inform effective prevention strategies. Among these, vascular health, particularly blood pressure, has garnered growing attention due to its significant impact on cerebrovascular integrity and brain function.^1^^,^^3^, ^4^, ^5^ Previous research from the Atherosclerosis Risk in Communities (ARIC) Study, the Framingham Offspring Study, the Multi-Ethnic Study of Atherosclerosis (MESA), the Systolic Blood Pressure (SBP) Intervention Trial, and the Women's Health Initiative (WHI), including our early reports, has shown that elevated SBP is associated with accelerated cognitive decline and increased risk of mild cognitive impairment and probable dementia.^4^^,^^6^, ^7^, ^8^, ^9^, ^10^ However, evidence on sex-specific differences in these associations remain limited and inconsistent.^11^, ^12^, ^13^, ^14^ While some studies suggest that women may be more susceptible to the adverse cognitive effects of elevated blood pressure, others report no significant variation based on sex. In addition, the apolipoprotein E (APOE) ε4 allele, a well-established genetic risk factor for late-onset Alzheimer's disease, has been shown to interact with vascular risk factors to accelerate neurodegeneration via mechanisms involving amyloid deposition, neuroinflammation, and vascular dysfunction.^15^, ^16^, ^17^ However, whether these interactions differ by sex remains under debate.^18^^,^^19^ A better understanding of sex-specific associations, interaction, and joint effects of vascular and APOE ε4 status on cognitive decline and dementia may provide critical insight for targeted prevention. Using data from the ARIC study,^20^ we investigated sex differences in the associations of vascular factors, including SBP, diastolic blood pressure (DBP), pulse pressure, ankle pressure, brachial pressure, and the ankle-brachial index (ABI), with cognitive decline and incident dementia. We also examined the joint and interactive effects of sex, APOE ε4, and vascular factors on the risk of dementia.

## Methods

### Study population

The ARIC study is a multi-site, prospective, community-based biracial cohort study funded by the National Heart, Lung, and Blood Institute (NHLBI) of the National Institutes of Health (NIH) in the United States,^20^ designed to investigate the etiology and clinical outcomes of atherosclerosis. The study initially enrolled a prospective cohort of 15,792 adults at baseline (1987 and 1989) from four U.S. communities: Forsyth County, North Carolina; Jackson, Mississippi; Minneapolis, Minnesota; and Washington County, Maryland, who underwent a comprehensive clinical examination. Within the cohort, 14,811 individuals were aged 45–64 years. Details of the baseline visit have been described previously. Cognitive function measurements in 14,348 participants began at Visit 2 (1990–1992), providing as baseline cognitive data in the ARIC study.^21^^,^^22^ Cases of dementia were ascertained annually through December 2019. The ARIC study was approved by institutional review boards (IRB) at all participating sites and all participants provided written informed consent. Data analyzed in our study were obtained from the NHLBI's Biospecimen and Data Repository Information Coordinating Center (RMDA No. 13661) and were approved by the Drexel University IRB (No. 2308010042).

### Participant selection in the analysis

**Fig. 1:**
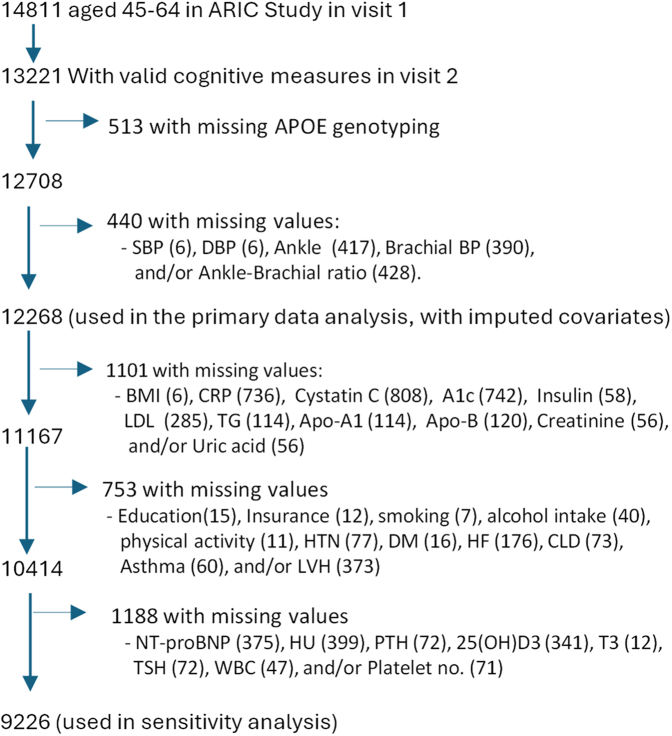
Sample flowchart. An individual may have multiple missing data. BMI, Body mass index; CRP, C-reactive protein; A1c, Hemoglobin A1c; LDL, Low density lipoprotein cholesterol; TG, Triglycerides; Apo A1, Apolipoprotein A1; Apo-B, Apolipoprotein B; HTN, Hypertension; DM, Diabetes; HF, Heart failure; CLD, Chronic lung disease; LVH, Left ventricular hypertrophy; NT-proBNP, N-terminal pro B-type natriuretic peptide; HU, Hormone use; PTH, Parathyroid hormone; 25(OH)D3, 25-hydroxyvitamin D3; T3, Thyroid hormone (T3: Triiodothyronine); TSH, Thyroid-Stimulating Hormone; WBC, White blood cell.

In the analysis, we included participants with complete data for the study exposures (blood pressure metrics and APOE genotyping) and outcomes (cognitive function measures and tracked incidence of dementia during the follow-up). Of 13,221 participants with valid cognitive function measures, we excluded 513 participants without APOE genotyping and 440 participants with any missing measures of blood pressure metrics (SBP = 6, DBP = 6, ankle pressure = 417, brachial pressure = 390, and/or ABI = 428), resulting in a final primary analysis sample of 12,268 participants. Fig. 1 presents the sample flowchart detailing the inclusion and exclusion criteria. A subset of 9266 participants, after excluding those with any missing values in covariates (see Fig. 1 for detail), was used for the non-imputed sensitivity analysis.

### Assessment of cognitive function

The ARIC study assessed cognitive function, which started in 1990–1992. Three neuropsychological tests were used to assess cognitive function: (1) the Digit Symbol Substitution Test (DSST), from the Wechsler Adult Intelligence Scale-Revised (WAIS-R), which assesses processing speed and executive function; (2) the Delayed Word Recall Test (DWRT), which evaluates verbal memory; and (3) the Word Fluency Test (WFT), also known as the Controlled Oral Word Association Test, which measures verbal fluency and executive function. Trained examiners administered these neuropsychological tests in a quiet room, following a standardized order during a single session.^22^ For data analysis, z-scores were computed for each cognitive test to account for their different scales, distributions, and demographic biases. This standardization ensured each test contributed equally to the analysis, preventing a single test with a wider numeric range from dominating the results. By placing all tests (DSST, DWRT, and WFT) on a common, adjusted scale, z-scores allow for fair comparison, combination, and interpretation in the ARIC's statistical analyses.^23^^,^^24^ A global cognitive z-score was then computed as the average of the individual DSST, DWRT, and WFT z-scores. Higher z-scores indicate better performance. For the purpose of this analysis, cognitive decline was defined as having cognitive z-scores falling within the lowest quintile (bottom 20%) of the distribution for each specific z-score metric.

### Assessment of incident dementia

Incident dementia cases were ascertained using a combination of neurocognitive testing, clinical adjudication, ICD-9 and ICD-10 hospital discharge codes, and death certificate codes. This process was further strengthened through the ARIC Neurocognitive Study beginning at the ARIC study Visit 5 (2011–2013). Adjudicated dementia cases were identified based on in-person cognitive testing, comprehensive neuropsychological assessments, and structured informant interviews. For participants who did not attend in-person visits, a modified telephone interview for cognitive status and informant interview were conducted to determine dementia status.^6^^,^^25^^,^^26^ Incident cases of dementia were tracked through December 2019 for all participants (n = 12,268).

### Exposure variables

Baseline blood pressure (Visit 1) was measured three times in a seated position using a random-zero sphygmomanometer after a 5-min rest. The average of the final two readings was used to calculate SBP, DBP, and pulse pressure (PP = SBP–DBP). Ankle pressure was measured using A DINAMAP™ 1846 SX automated oscillometric device (Critikon, Tampa, FL).^27^ Brachial pressure was measured in the supine position during carotid artery scanning and was taken automatically every 5 min. Ankle-brachial index was calculated as the average of the two resting ankle pressure readings divided by the average of the first two resting brachial pressure readings. Hypertension was classified into four categories based on the 2017 American Heart Association guidelines: (1) Normal blood pressure: SBP <120 mm Hg and DBP <80 mm Hg; (2) Elevated blood pressure: SBP 120–129 mm Hg and DBP<80 mm Hg; (3) Hypertension Stage 1: SBP 130–139 mm Hg or DBP 80–89 mm Hg; and (4) Hypertension Stage 2: SBP ≥140 mm Hg or DBP ≥90 mm Hg, or use of antihypertensive medications.^28^ APOE genotyping was performed using the TaqMan assay (Applied Biosystems, Foster City, CA),^29^ and categorized as 0, 1 or 2 copies of the APOE ε4 allele.

### Covariates

Multivariable regression models were adjusted for baseline key covariates to account for potential confounding. These included age (this was specifically included in the analysis of blood pressure metrics and cognitive decline), race/ethnicity (African Americans and Whites), educational attainment (less than high school, high school graduate or high school equivalent or vocational school, and college or higher), smoking status (never, former, and current), body mass index (BMI, kg/m^2^), serum high-sensitivity C-reactive protein (CRP, mg/L), and Cystatin C (mg/L). Missing values in covariates which were adjusted in multivariable models were imputed. In the imputation process, continuous variables (BMI, CRP, and Cystatin C) were handled using Markov Chain Monte Carlo (MCMC) methods, and categorical variables (education and smoking status) employed the Missing Indicator Method.^30^^,^^31^

### Statistical analysis

We conducted five sets of analyses. (1) Participants' characteristics were compared by sex. Differences in means for continuous variables were tested using Student's *t*-tests, and differences in proportions for categorical variables using Chi-square tests. (2) Quantile–Quantile (Q–Q) plots were used to asses assumptions for the linear regression of four blood pressure metrics (SBP, pulse pressure, ankle pressure, and brachial pressure) on cognitive z-scores. We then fitted sex-specific linear regression models to examine the association of SBP, pulse pressure, ankle and brachial pressures with four cognitive function z-scores (global cognitive, DSST, DWRT, and WFT z-scores). LOWESS regression was employed to investigate and reveal the non-linear association between the ankle-brachial index (ABI) and cognitive decline. We then added a quadratic term for ABI to the linear regression models. Additionally, a restricted cubic spline regression model was applied to further account for this non-linear relationship. In multivariable linear regression analyses, two adjusted models were performed. Model 1 was adjusted for age and race/ethnicity. Model 2, the full model, was further adjusted for education, BMI, smoking status, CRP, Cystatin C, and APOE ε4. We did not adjust chronic conditions (diabetes mellitus and cardiovascular diseases (coronary heart disease, heart failure, or stroke) because they are considered potential mediators of the relationship between blood pressure metrics and cognitive declines. (3) Cox proportional hazards regression models were used to examine the longitudinal association between blood pressure metrics and dementia risk. Follow-up time was calculated in years from each participant's baseline entry date to the date of their dementia diagnosis, or death, or the end of the study follow-up (December 31, 2019), whichever occurred first. Participants with baseline prevalent dementia were excluded in this analysis. The proportional hazards assumption was tested using Schoenfeld residuals and visual examinations of “log–log” plots.^32^^,^^33^ To estimate hazards ratios (HR) of dementia, blood pressure metrics (SBP, pulse pressure, ankle and brachial pressures) were categorized into quintiles (Q1–Q5), with the highest quintile (Q5) defined as elevated blood pressure level. The ABI was classified into three groups: decreased (ABI <1), normal (ABI: 1–1.2), and elevated (ABI ≥1.3). This categorization was based on its non-linear relationship with cognitive decline and its clinical application. In the longitudinal analysis, age was used as the time scale (years) in Cox regression models. This approach has an advantage to better account for the effects of aging on the hazard of a disease strongly correlated to age and helps to reduce bias, particularly in observational studies where participants enter the study at different ages (left truncation).^34^ (4). We estimated the joint effects of sex and blood pressure metrics on dementia risk using the total sample. We then evaluated the joint-effects of the APOE ε4 allele and blood pressure metrics on dementia risk separately for women and men. To quantify the difference between sexes, we calculated relative excess risk (RER, %) of dementia attributable to the presence of the APOE ε4 allele and elevated blood pressure metrics in women vs. men using the formula: Relative excess risk (%) = [(HR1–HR2)/(HR1-1)] × 100, where HR1 is the hazard ratio of dementia in women and HR2 in men.^35^ (5). Interaction effects of sex and blood pressure metrics (assigned as sex [men = 0 and women = 1] x blood pressure metrics [in binary]) and of sex and APOE ε4 allele (≥1 copy of ε4 alleles vs. zero copy, and 2 copies vs. zero) on dementia risk were examined using the total sample, and interaction effects of APOE ε4 allele and blood pressure metrics (elevated vs. non-elevated) were evaluated to assess whether sex or APOE ε4 modified the associations between blood pressure metrics and dementia risk.

We conducted three sensitivity analyses: (1). Comparison with complete case analysis: We restricted the analysis sample to only those participants without any missing values and without imputation (n = 9266). (2) To assess the robustness of our findings, we excluded dementia cases diagnosed within the first 12 months of follow-up. This approach reduced potential overestimation of hazards ratios due to undetected or pre-existing dementia at baseline. (3). To address the potential inflation of Type I error rates attributable to multiple comparisons in significant tests (for variables with ≥3 groups), we used the Holm–Bonferroni method to adjust the p-values.^36^ This procedure was applied to these variables: (a) SBP/DBP (in four groups: <120/<80, 120–129/<80, 130–139/80–89, and ≥140/≥90 mm Hg); (b) Ankle to brachial index (in three groups: decreased, normal, and increased; and (c) APOE ε4 (in three groups: by the number of ε4 copies, 0–2).

All statistical analyses were performed using SAS software, version 9.4 (SAS Institute Inc., Cary, NC). A 2-sided p-value of <0.05 was considered as statistically significant.

### Role of the funding source

The funders of the study had no role in study design, data collection, data analysis, data interpretation, or writing of the manuscript.

## Results

### Characteristics of the study participants

Table 1 shows that baseline mean (SD) ages of the participants were 53.8 (5.6) in women and 54.5 (5.7) in men. Women had significantly lower means of SBP, DBP, ankle pressure, brachial pressure, ABI, and serum Cystatin C concentrations. In contrast, men had significantly lower BMI and CRP (log-transformed values). Significant sex differences were also observed in the distributions of race/ethnicity, education level, and smoking status. Women had a lower prevalence of coronary heart disease, but a higher prevalence of heart failure than men.

**Table 1:** Baseline characteristics of participants (n = 12,268).

	Women (n = 6751)		Men (n = 5517)		p-value
	Mean, no	(±SD), (%)	Mean, no	(±SD), (%)	
**Continuous var, mean (SD)**					
Age, year	53.8	(5.6)	54.5	(5.7)	**<0.0001**
SBP, mm Hg	119.5	(19.0)	121.5	(17.1)	**<0.0001**
DBP, mm Hg	71.9	(10.7)	75.1	(10.9)	**<0.0001**
Ankle BP, mm Hg	139.8	(24.1)	152.6	(22.9)	**<0.0001**
Brachial BP mm Hg	125.1	(21.0)	130.5	(18.3)	**<0.0001**
Ankle to brachial index	1.11	(0.1)	1.16	(0.1)	**<0.0001**
BMI, kg/m^2^	27.7	(6.0)	27.5	(4.1)	**0.035**
Log-CRP, mg/L	1.45	(0.8)	1.24	(0.7)	**<0.0001**
Cystatin C, mg/L	0.87	(0.3)	0.91	(0.2)	**<0.0001**
**Categorical var, no. (%)**					
African American	1771	(26.2)	1035	(18.8)	**<0.0001**
Whites	4980	(73.8)	4482	(81.2)	
Education, %					**<0.0001**
<High school	1422	(21.1)	1146	(20.8)	
High school	2581	(38.3)	1511	(27.4)	
>High school	2740	(40.6)	2852	(51.8)	
Smoking status					**<0.0001**
Never	3588	(53.2)	1605	(29.1)	
Former	1571	(23.3)	2488	(45.1)	
Current	1585	(23.5)	1423	(25.8)	
Chronic condition, no, %					
Hypertension	2384	(35.5)	1890	(34.6)	0.27
Diabetes	716	(10.7)	611	(11.1)	0.43
CHD	175	(2.6)	474	(8.6)	**<0.0001**
Heart failure	375	(5.6)	146	(2.7)	**<0.0001**
Stroke	383	(5.7)	270	(4.9)	0.06
APOE ε4 genotype, %					0.48
ε4 copy No. = 0	4688	(69.4)	3819	(69.2)	
ε4 copy No. = 1	1883	(27.9)	1568	(28.4)	
ε4 copy No. = 2	180	(2.7)	130	(2.4)	

BP, Blood pressure; SBP, Systolic BP; DBP, Diastolic BP; BMI, Body mass index; Log-CRP, Log value of C-Reactive Protein; CHD, Coronary heart disease. Statistical significance was defined as p < 0.05 (bold). All tests were two-tailed.

Supplementary Table S1 demonstrates that individuals with dementia in both men and women, were older and had higher SBP, DBP, ankle and brachial pressures compared to those without dementia. Notably, differences in these blood pressure metrics were more pronounced in women than in men.

**Fig. 2:**
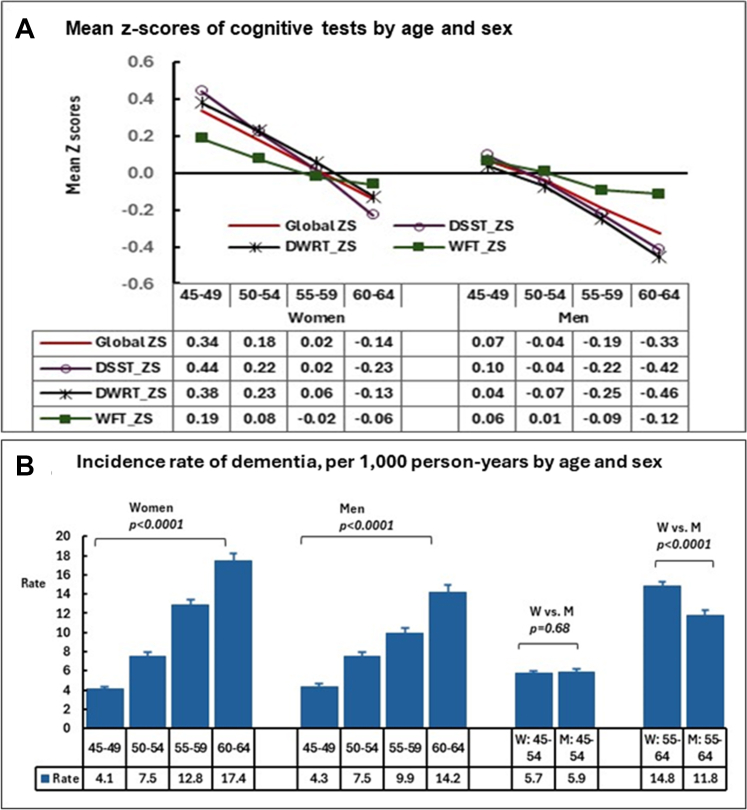
Mean Z-scores of cognitive tests (A) and incidence of dementia per 1000 person-years (B) by Ages in Women and Men. Global ZS, Global cogntive Z score; DSST ZS, Z score of digit symbol substitution test; DWRT ZS, Z score of delayed word recall test; WFT ZS, Z score of word fluency test.

Fig. 2A shows that older age was significantly associated with lower mean z-scores for global cognitive function, DSST, DWRT, and WFT in both women and men.

### Incidence of dementia by age and sex

Among 12,268 adults aged 45–64 years followed over a median of 26.4 years (interquartile range [IQR]: 19.5–31.2 years), a total of 2698 participants developed dementia over 299,431 person-years of follow-up. Increased ages were significantly associated with an increased risk of dementia in both sexes. In participants aged 45–54 years, the dementia incidence rates were similar between women and men (5.7 vs. 5.9 per 1000 person-years, p = 0.68). However, among those aged 55–64, women had a significantly higher incidence rate of dementia (14.8 [95% CI: 13.9–15.7] per 1000 person-years), compared to men (11.8 [95% CI: 11.0–12.7] per 1000 person-years), rate difference, p < 0.0001 (Fig. 2B).

### Association between vascular factors and cognitive function by sex

Table 2 details the association between blood pressure metrics and cognitive function measures. In Model 1 (adjusted for age and race), women showed a linear association of SBP, pulse pressure, ankle and brachial pressures with declines in global cognitive function, DSST, DWRT, and WFT z-scores. In men, elevated SBP, pulse pressure, ankle and brachial pressures were associated with decreased global cognitive function, DSST, DWRT, and WFT z-scores. In contrast, the ankle-brachial index (ABI) was nonlinearly associated with DSST score (regression coefficient = −0.869, p = 0.0044) in women, and with global cognitive z-score (β = −0.752, p = 0.0047), DSST (β = −0.642, p = 0.033), and WFT z-score (β = −0.911, p = 0.020) in men. In Model 2 (the fully adjusted models), although the associations were attenuated, yet significant findings remained predominantly in women. In men, only a few specific associations persisted: higher pulse pressure was associated with declines in global cognitive z-score (p = 0.021) and DSST z-score (p = 0.0016), and higher ankle pressure was associated with declines in global cognitive function (p = 0.010) and WFT (p = 0.0041). Furthermore, the significant non-linear association of ABI with global cognitive function and WFT z-scores shitted to men only in the fully adjusted models (Model 2).

**Table 2:** Association of SBP, ankle and brachial pressures with risk of cognitive decline by sex.

BP associated with cognitive decline	Women (N = 6751)						Men (N = 5517)					
	Model 1			Model 2			Model 1			Model 2		
	β	(SE)	p-value	β	(SE)	p-value	β	(SE)	p-value	β	(SE)	p-value
SBP per 10 mm Hg												
Global ZS	−0.037	(0.004)	**<0.0001**	−0.016	(0.004)	**0.0001**	−0.014	(0.006)	**0.014**	−0.008	(0.005)	0.10
DSST_ZS	−0.036	(0.005)	**<0.0001**	−0.013	(0.005)	**0.0097**	−0.011	(0.006)	0.07	−0.006	(0.006)	0.31
DWRT_ZS	−0.027	(0.006)	**<0.0001**	−0.016	(0.006)	**0.015**	−0.015	(0.008)	0.06	−0.010	(0.008)	0.20
WFT_ZS	−0.049	(0.006)	**<0.0001**	−0.018	(0.006)	**0.0034**	−0.015	(0.008)	0.07	−0.009	(0.008)	0.23
PP per 10 mm Hg												
Global ZS	−0.059	(0.006)	**<0.0001**	−0.028	(0.005)	**<0.0001**	−0.029	(0.008)	**0.0002**	−0.016	(0.007)	**0.021**
DSST_ZS	−0.053	(0.007)	**<0.0001**	−0.018	(0.007)	**0.0060**	−0.040	(0.009)	**<0.0001**	−0.024	(0.008)	**0.0016**
DWRT_ZS	−0.050	(0.009)	**<0.0001**	−0.033	(0.009)	**0.0001**	−0.020	(0.011)	0.07	−0.010	(0.011)	0.33
WFT_ZS	−0.074	(0.009)	**<0.0001**	−0.033	(0.008)	**<0.0001**	−0.026	(0.011)	**0.022**	−0.013	(0.010)	0.21
Ankle BP per 10 mm Hg												
Global ZS	−0.029	(0.003)	**<0.0001**	−0.014	(0.003)	**<0.0001**	−0.005	(0.004)	0.24	−0.009	(0.004)	**0.010**
DSST_ZS	−0.027	(0.004)	**<0.0001**	−0.012	(0.004)	**0.0035**	0.002	(0.005)	0.73	−0.005	(0.004)	0.19
DWRT_ZS	−0.017	(0.005)	**0.001**	−0.010	(0.005)	**0.045**	−0.005	(0.006)	0.39	−0.007	(0.006)	0.23
WFT_ZS	−0.041	(0.005)	**<0.0001**	−0.019	(0.005)	**<0.0001**	−0.011	(0.006)	0.06	−0.016	(0.006)	**0.0041**
Brachial BP per 10 mm Hg												
Global ZS	−0.042	(0.004)	**<0.0001**	−0.017	(0.004)	**<0.0001**	−0.016	(0.005)	**0.0016**	−0.007	(0.005)	0.15
DSST_ZS	−0.040	(0.005)	**<0.0001**	−0.013	(0.005)	**0.0060**	−0.010	(0.006)	0.08	0.000	(0.005)	0.93
DWRT_ZS	−0.031	(0.006)	**<0.0001**	−0.018	(0.006)	**0.0024**	−0.019	(0.007)	**0.0099**	−0.011	(0.007)	0.12
WFT_ZS	−0.056	(0.006)	**<0.0001**	−0.021	(0.006)	**0.0001**	−0.020	(0.008)	**0.0081**	−0.009	(0.007)	0.20
ABI in quadratic												
Global ZS	−0.464	(0.251)	0.06	−0.154	(0.224)	0.49	−0.752	(0.266)	**0.0047**	−0.659	(0.233)	**0.0048**
DSST_ZS	−0.869	(0.305)	**0.0044**	−0.505	(0.275)	0.07	−0.642	(0.300)	**0.033**	−0.486	(0.262)	0.06
DWRT_ZS	−0.398	(0.356)	0.26	−0.161	(0.351)	0.65	−0.703	(0.373)	0.06	−0.654	(0.367)	0.04
WFT_ZS	−0.126	(0.364)	0.73	−0.154	(0.224)	0.49	−0.911	(0.391)	**0.020**	−0.659	(0.233)	**0.0048**

BP, Blood pressure; SBP, Systolic blood pressure; PP, Pulse pressure; ABI, Ankle–brachial index; Global ZS, Mean of DSST_ZS, DWRT_ZS, and WFT_ZS. DSST_ZS: Z score of digit symbol substitution test; DWRT_ZS, Z score of delayed word recall test; WFT_ZS, Z score of word fluency test. Model 1. Adjusted for age and race/ethnicity. Model 2: Adjusted for age, race/ethnicity, education, body mass index (BMI), smoking, Cystatin C, CRP, and APOE gene. Statistical significance was defined as p < 0.05 (bold). All tests were two-tailed.

### Hazards ratios of dementia associated with blood pressure metrics by sex

In Table 3, Model 1 shows that individuals with hypertension (SBP/DBP ≥140/90 mm Hg), elevated brachial pressure, and those carrying the APOE ε4 allele in women, and in those with hypertension, elevated brachial pressure, decreased ABI, and those carrying the APOE ε4 allele in men had a significantly higher risk of dementia. Although the magnitudes of these associations were attenuated after further adjustment for covariates in Model 2, these associations remained significant in both women and men, except for a bordline significant association in hypertension in men (p = 0.06). A notable result is the pronounced sex difference in dementia risk associated with the APOE ε4 allele, with women facing a substantially higher risk in those with two copies of APOE ε4 allele (HR = 4.39, 95% CI: 3.52–5.47) than men (HR = 2.92, 95% CI: 2.20–3.88).

**Table 3:** Hazards ratio (95% CI) of dementia associated with vascular factors and APOE ε4 by sex.

BP Metrics, mmHg	Women						Men					
	Model 1			Model 2			Model 1			Model 2		
	HR	(95% CI)	p-value	HR	(95% CI)	p-value	HR	(95% CI)	p-value	HR	(95% CI)	p-value
SBP/DBP												
<120/<80	1			1			1			1		
120–129/<80	1.09	(0.91–1.31)	0.34	1.05	(0.87–1.26)	0.61	1.02	(0.82–1.26)	0.89	1.00	(0.80–1.24)	0.98
130–139/80–89	1.04	(0.87–1.25)	0.65	1.02	(0.85–1.22)	0.85	1.10	(0.91–1.33)	0.32	1.12	(0.93–1.35)	0.25
≥140/≥ 90	1.26	(1.12–1.42)	**<0.0001**	1.21	(1.07–1.36)	**0.0025**	1.18	(1.03–1.35)	**0.021**	1.15	(1.00–1.33)	0.06
Elevated SBP												
No	1			1			1			1		
Yes	1.03	(0.92–1.16)	0.60	1.02	(0.90–1.15)	0.75	0.97	(0.84–1.13)	0.73	0.96	(0.83–1.11)	0.58
Elevated PP												
No												
Yes	1.06	(0.95–1.18)	0.33	1.04	(0.93–1.16)	0.54	0.97	(0.83–1.13)	0.68	0.97	(0.83–1.13)	0.65
Elevated ankle BP												
No	1			1			1			1		
Yes	1.09	(0.95–1.24)	0.22	1.05	(0.92–1.20)	0.45	1.03	(0.90–1.17)	0.71	1.01	(0.89–1.15)	0.88
Elevated brachial BP												
No	1			1			1			1		
Yes	1.24	(1.10–1.39)	**0.0005**	1.14	(1.01–1.29)	**0.034**	1.29	(1.13–1.49)	**<0.0001**	1.24	(1.08–1.43)	**0.0024**
ABI in group												
Decreased	0.97	(0.85–1.11)	0.65	0.91	(0.80–1.04)	0.16	1.55	(1.26–1.90)	**<0.0001**	1.53	(1.25–1.89)	**<0.0001**
Normal	1			1			1			1		
Elevated	1.02	(0.86–1.21)	0.82	1.01	(0.85–1.20)	0.92	0.93	(0.78–1.10)	0.38	0.96	(0.81–1.15)	0.67
APOE ε4 genotype, %												
ε4 copy no. = 0	1			1			1			1		
ε4 copy no. = 1	1.77	(1.60–1.97)	**<0.0001**	1.78	(1.61–1.98)	**<0.0001**	1.66	(1.47–1.89)	**<0.0001**	1.69	(1.49–1.92)	**<0.0001**
ε4 copy no. = 2	4.31	(3.46–5.37)	**<0.0001**	4.39	(3.52–5.47)	**<0.0001**	2.79	(2.11–3.71)	**<0.0001**	2.92	(2.20–3.88)	**<0.0001**

BP, Blood pressure; HR, Hazards ratio; SBP/DBP, Systolic/diastolic blood pressure; PP, Pulse pressure; ABI, Ankle–brachial index; APOE, Apolipoprotein E.

Hazard ratios (HRs) of dementia associated with blood pressure metrics were estimated by using baseline age plus follow-up year as time scale for more robustly controlling age effect on the study outcomes. Model 1. Adjusted race/ethnicity and education. Model 2: Adjusted race/ethnicity, education, BMI, smoking. Cystatin C, CRP, and APOE gene. Model 2: Adjusted for race/ethnicity, education, body mass index, smoking, Cystatin C, CRP, and APOE gene. In Model 1 and Model 2 when examining association between APOE e4 and risk of dementia, APOE gene was not adjusted because it was the exposure. Statistical significance was defined as p < 0.05 (bold). All tests were two-tailed.

### Absolut effects of sex combined APOE ε4 and blood pressure metrics on dementia risk

Table 4 presents the absolute effects of sex combined APOE ε4 and blood pressure metrics on dementia risk by keeping males without the exposures as the comparison group for both sexes. Model 1 indicates that APOE ε4 significantly increased dementia risk for both sexes. In the fully adjusted model, the absolute risk of dementia for exposed women and exposed men is nearly identical (HR 1.77 vs. 1.79). Hypertension significantly increased the absolute risk of dementia for men (HR 1.14, p = 0.032). In contrast, the risk for women with hypertension is not significantly elevated above the unexposed male (HR 1.09, p = 0.16). Elevated (E)-brachial pressure confers a significantly higher absolute risk of dementia for men (HR 1.20, p = 0.0086).

**Table 4:** Effect of sex combined with APOE ε4 and blood pressure metrics on risk of dementia (men without these risk exposures as the reference group for men and women).

Effect of exposures		Race-adjusted			Fully adjusted HR		
		HR	(95% CI)	p-value	HR	(95% CI)	p-value
Sex	APOE e4						
Men	No	1			1		
Men	Yes	1.75	(1.55–1.97)	**<0.0001**	1.79	(1.59–2.02)	**<0.0001**
Women	No	0.95	(0.86–1.05)	0.31	0.92	(0.83–1.02)	0.11
Women	Yes	1.82	(1.63–2.04)	**<0.0001**	1.77	(1.58–1.98)	**<0.0001**
Sex	HTN						
Men	No	1			1		
Men	Yes	1.18	(1.04–1.33)	**0.0083**	1.14	(1.01–1.29)	**0.032**
Women	No	0.94	(0.84–1.04)	0.21	0.92	(0.82–1.02)	0.11
Women	Yes	1.18	(1.06–1.31)	**0.0033**	1.09	(0.97–1.22)	0.16
Sex	E-SBP						
Men	No	1			1		
Men	Yes	0.99	(0.86–1.14)	0.88	0.96	(0.83–1.11)	0.61
Women	No	0.95	(0.87–1.03)	0.23	0.92	(0.84–1.01)	0.07
Women	Yes	1.02	(0.90–1.16)	0.75	0.94	(0.83–1.07)	0.36
Sex	E-PP						
Men	No	1			1		
Men	Yes	0.98	(0.84–1.14)	0.82	0.95	(0.82–1.11)	0.50
Women	No	0.94	(0.86–1.03)	0.17	0.92	(0.84–1.00)	0.06
Women	Yes	1.03	(0.92–1.16)	0.62	0.94	(0.84–1.07)	0.35
Sex	E− Ankle BP						
Men	No	1			1		
Men	Yes	1.04	(0.91–1.18)	0.59	1.03	(0.90–1.17)	0.68
Women	No	0.96	(0.87–1.05)	0.32	0.93	(0.85–1.02)	0.13
Women	Yes	1.08	(0.94–1.24)	0.26	0.99	(0.86–1.14)	0.89
Sex	E-Brachial BP						
Men	No	1			1		
Men	Yes	1.24	(1.08–1.42)	**0.0027**	1.20	(1.05–1.38)	**0.0086**
Women	No	0.98	(0.90–1.07)	0.63	0.95	(0.87–1.05)	0.31
Women	Yes	1.17	(1.03–1.33)	**0.017**	1.06	(0.93–1.21)	0.41

In Cox model: Age was used as the time scale for control age effect. HR, Hazards ratio; E-SBP, Elevated systolic BP; E-PP, Elevated pulse pressure; E-Ankle BP, Elevated ankle BP; E-Brachial BP, Elevated Brachial BP.

Fully adjusted HR: Adjusted for race, education, BMI, smoking, cystatin C, and CRP. APOE e4 in two groups (ε4 ≥1 copies vs. without ε4 carry). Statistical significance was defined as p < 0.05 (bold). All tests were two-tailed.

### Joint effect of the APOE ε4 allele and blood pressure metric on dementia risk by sex

Table 5 shows that the magnitude of the risk increases attributable to the joint exposures (APOE ε4 plus a blood pressure metric) was almost consistently higher in women than in men. For instance, the joint effect of APOE ε4 (≥1 copy) and hypertension (yes vs. no) resulted in a HR of 2.26 (95% CI: 1.95–2.63) in women compared the women without these exposures. The corresponding HR was 1.91 (95% CI: 1.59–2.29) in men. This difference is quantified by the relative excess risk (RER, %), which was 28% (RER: [2.26–1.91]/[2.26–1] x 100). Across all blood pressure metrics examined, the RER for women compared to men ranged from a low of 23% (APOE ε4 plus elevated ankle pressure) to a high of 46% (APOE ε4 plus elevated pulse pressure).

**Table 5:** Joint effect of vascular factors and APOE ε4 on risk of dementia by sex.

Joint effect		Women			Men			% of RER in women vs. men
		HR	(95% CI)	p-value	HR	(95% CI)	p-value	
APOE ε4	HTN							
No	No	1			1			
No	Yes	1.25	(1.09–1.43)	**0.0010**	1.22	(1.04–1.43)	**0.015**	
Yes	No	2.05	(1.78–2.35)	**<0.0001**	1.94	(1.65–2.27)	**<0.0001**	
Yes	Yes	2.26	(1.95–2.63)	**<0.0001**	1.91	(1.59–2.29)	**<0.0001**	28
APOE ε4	SBP (Q5)							
No	No	1			1			
No	Yes	1.03	(0.88–1.20)	0.74	1.02	(0.84–1.23)	0.85	
Yes	No	1.94	(1.73–2.18)	**<0.0001**	1.83	(1.60–2.10)	**<0.0001**	
Yes	Yes	1.96	(1.64–2.34)	**<0.0001**	1.61	(1.29–2.02)	**<0.0001**	36
APOE ε4	PP (Q5)							
No	No	1			1			
No	Yes	0.93	(0.80–1.08)	0.36	1.01	(0.83–1.23)	0.90	
Yes	No	1.81	(1.61–2.04)	**<0.0001**	1.81	(1.59–2.08)	**<0.0001**	
Yes	Yes	2.16	(1.84–2.55)	**<0.0001**	1.63	(1.28–2.07)	**<0.0001**	46
APOE ε4	Ankle BP (Q5)							
No	No	1			1			
No	Yes	1.15	(0.97–1.36)	0.12	1.11	(0.94–1.31)	0.24	
Yes	No	2.00	(1.79–2.24)	**<0.0001**	1.90	(1.64–2.19)	**<0.0001**	
Yes	Yes	1.88	(1.54–2.29)	**<0.0001**	1.68	(1.38–2.05)	**<0.0001**	23
APOE ε4	Brachial BP (Q5)							
No	No	1			1			
No	Yes	1.18	(1.01–1.39)	**0.040**	1.30	(1.08–1.56)	**0.0046**	
Yes	No	2.01	(1.79–2.25)	**<0.0001**	1.86	(1.62–2.14)	**<0.0001**	
Yes	Yes	1.99	(1.66–2.38)	**<0.0001**	1.99	(1.61–2.47)	**<0.0001**	–

RER: Relative excess risk %. In Cox model: Age was used as the time scale for control age effect. HR: Hazards ratio. HR is adjusted for race/ethnicity, education, body mass index, smoking, cystatin C, and CRP. HTN: Hypertension, defined as SBP/DBP ≥140/90 mm Hg or current use of antihypertensive medication. Elevated ankle blood pressure (BP) and elevated brachial BP are defined as values in the highest 20% (Q5). Relative excess risk (RER, %) is estimated using the formula: (HR1−HR2)/(HR1−1) × 100, where HR1 is in women, and HR2 in men. Statistical significance was defined as p < 0.05 (bold). All tests were two-tailed.

### Testing interaction effects

**Fig. 3:**
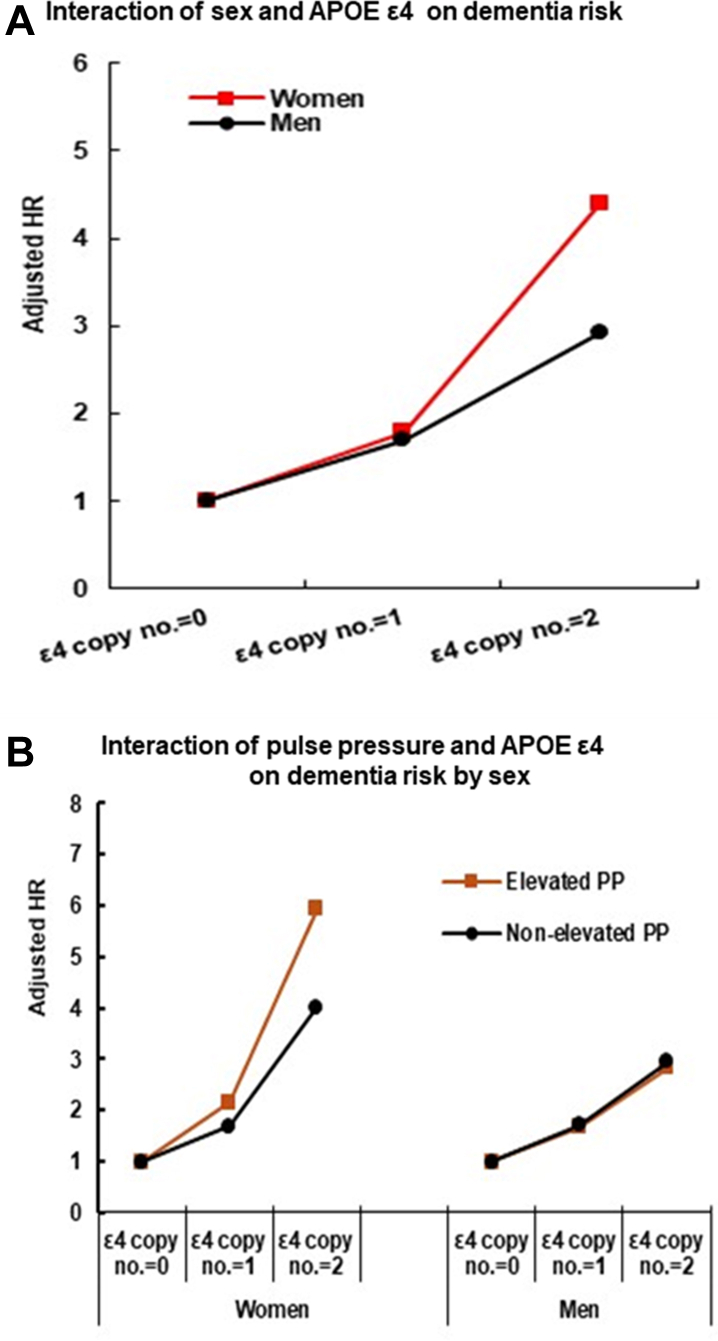
Sex–Specific association of APOE ε4 copy number and pulse pressure with dementia risk. Note: **Panel A:** The lines illustrate the adjusted Hazard Ratios (HR) for dementia in women (red squares) and men (black circles) compared to individuals with zero copies of the APOE ε4 allele (reference HR = 1.0). The multiplicative interaction of sex × APOE ε4 (copy number 2 vs. 0) was statistically significant (HR = 1.48, 95% CI: 1.10−2.0). **Panel B** illustrates the adjusted Hazard Ratios (HR) for dementia associated with APOE ε4 copy number, stratified by elevated pulse pressure (PP) (brown squares) and non-elevated pulse pressure (black circles). **In Women:** A significant multiplicative interaction was observed between APOE ε4 copy number and elevated PP (HR: 1.28, 95% CI: 1.02−1.61). **In Men:** No statistically significant multiplicative interaction was observed between APOE ε4 copy number and elevated PP (p = 0.45).

Table 6 shows that in the total sample, we did not observe statistically significant multiplicative interaction between sex and blood pressure metrics. However, a significant interaction was observed between sex (women vs. men) and carrying two copies of APOE ε4 allele (vs. zero copy) on the risk of dementia (HR: 1.48, 95% CI: 1.10–2.00, p = 0.0094). This indicates that the effect of carrying two APOE ε4 alleles on dementia risk was 48% greater in women compared to men (Table 6 and Fig. 3A). In the sex-stratified analysis (Fig. 3B), a significant interaction was found between APOE ε4 carrier status and elevated pulse pressure (p = 0.032) in women. The interaction HR (1.28, 95% CI: 1.02–1.61) suggests that the effect of APOE ε4 carriage on dementia risk is 28% greater when combined with elevated pulse pressure compared to non-elevated pulse pressure (Fig. 3B). No significant multiplicative interactions were observed in men between APOE ε4 carriage and elevated pulse pressure, suggesting that the effect of APOE ε4 carriage does not vary substantially based on pulse pressure status.

**Table 6:** Interaction of sex × APOE ε4 in all sample, and APOE ε4 × blood pressure metrics by sex on risk of dementia.

	HR	(95% CI)	p-value
**In all sample**			
SEX × HTN	1.04	(0.89–1.21)	0.64
SEX × SBP	1.06	(0.88–1.28)	0.53
SEX × PP	1.08	(0.90–1.31)	0.40
SEX × Ankle BP	1.04	(0.86–1.24)	0.71
SEX × Brachial BP	0.92	(0.77–1.11)	0.38
SEX × APOE ε4 (no. copy 1 vs. 0)	1.08	(0.92–1.26)	0.36
SEX × APOE ε4 (no. copy 2 vs. 0)	1.48	(1.10–2.00)	**0.0094**
**In women**			
APOE ε4 × HTN	0.89	(0.72–1.08)	0.23
APOE ε4 × SBP	0.98	(0.78–1.25)	0.89
APOE ε4 × PP	1.28	(1.02–1.61)	**0.032**
APOE ε4 × Ankle BP	0.82	(0.63–1.06)	0.13
APOE ε4 × Brachial BP	0.84	(0.66–1.07)	0.15
**In men**			
APOE ε4 × HTN	0.81	(0.63–1.03)	0.08
APOE ε4 × SBP	0.86	(0.64–1.16)	0.33
APOE ε4 × PP	0.89	(0.65–1.21)	0.45
APOE ε4 × Ankle BP	0.80	(0.62–1.04)	0.10
APOE ε4 × Brachial BP	0.82	(0.62–1.09)	0.18

Hazard ratios (HRs) were estimated by using baseline age plus follow-up year as time scale for more robustly controlling age effect on the study outcomes. HTN, Hypertension; SBP, Systolic BP; PP, Pulse blood pressure. Test interaction: Adjusted for race/ethnicity, education, BMI, smoking, Cystatin C, and CRP. Statistical significance was defined as p < 0.05 (bold). All tests were two-tailed.

We performed three distinct sensitivity analyses: (1) We repeated the analysis using the non-imputed sample (n = 9266). The overall results were consistent with the main findings from the imputed sample (n = 12,268). Elevated blood pressure metrics remained significantly associated with declines in cognitive function z-scores for both sexes (Model 1 in Supplementary Table S2). Compared to the primary analysis, the associations were attenuated in the fully adjusted models (model 2), particularly for men, highlighting the influence of potential confounding factors and the impact of reduced statistical power within the smaller sample. The overall associations of blood pressure metrics and APOE ε4 allele with dementia risk in the non-imputed sample (Supplementary Table S3) were consistent with those observed in the imputed analysis. (2) To minimize the potential for reverse causation bias (where pre-clinical dementia affects baseline blood pressure measurements), we excluded dementia cases diagnosed within the first 12 months of follow-up in the longitudinal analysis. This exclusion did not significantly change the main results and conclusions. (3) To rigorously account for multiple comparisons, we applied the Holm–Bonferroni method using PROC MULTTEST in SAS to estimate adjusted p-values (coaction p-values). This correction was specifically performed for categorical variables with three or more groups (SBP/DBP, ABI, and APOE ε4 allele in groups). Although these estimated p-values became more conservative after applying the Holm–Bonferroni tests, the key associations remained statistically significant (p < 0.05 or p < 0.01), confirming the reliability of our findings.

## Discussion

Over a median follow-up of 26.4 (IQR: 19.5–31.2) years, the main findings of this study demonstrated significant sex-differences in the relationship between blood pressure metrics and cognitive declines. The key results highlight a stronger vascular risk burden in women: (1) Vascular risk factors, including hypertension, elevated SBP, pulse pressure, ankle and brachial pressures, were significantly associated with cognitive decline and dementia risk in women. In contrast, these associations were generally weaker or attenuated in men. (2) A significant joint effect of blood pressure metrics and APOE ε4 allele on dementia risk was observed in both sexes. However, the relative excess risk conferred by this combination was notably greater in women compared to men, ranging from 23% to 46% higher depending on the specific blood pressure metrics. (3) The Ankle-Brachial Index (ABI) showed a significant nonlinear association with global cognitive and WFT z-scores in men but not in women (in the fully adjusted model). These findings underscore a critical sex-based dimorphism in vascular vulnerability to dementia and suggest that women may be disproportionately affected by cumulative burden of genetic susceptibility and vascular risk factors.

Several epidemiological studies have investigated the associations between vascular factors and cognitive outcomes.^8^^,^^25^^,^^37^^,^^38^ Early findings from the ARIC study explored the associations of hypertension and arterial stiffness with cognitive impairment and dementia in both sexes.^6^^,^^14^^,^^39^ However, few studies have specifically focused on sex-based comparisons. The Women's Health Initiative Memory Study (WHIMS), including our own prior work, demonstrated significant associations between elevated SBP and cognitive decline in postmenopausal women.^4^^,^^40^^,^^41^ While important, the generalizability of the WHIMS findings is limited due to its selected study population. The Framingham Heart Study has contributed valuable insights into how vascular risk factors, including SBP and pulse pressure relate to brain aging and dementia risk.^42^^,^^43^ Similarly, the MESA has enriched the literature by incorporating advanced imaging data to assess subclinical cardiovascular disease in relation to cognitive function,^44^ as well as our own prior report linking cardiometabolic factors to cognitive impairment from the MESA.^7^ Despite these contributions, a critical gap remains: most prior studies have not systematically tested for sex differences or examined an overall effect of vascular factors and APOE ε4 on dementia risk in a sample of combined both sexes. While some presented sex-stratified descriptive results, few performed rigorous joint and interaction testing. This oversight may obscure clinically meaningful heterogeneity, as men and women differ markedly in vascular aging trajectories, hormonal environments, inflammatory responses, and patterns of neurodegeneration. In 2024, the Lancet Commission on dementia prevention, based on findings from systematic reviews and meta-analyses, proposed that hypertension is one of twelve identified risk factors for dementia (i.e., less education, hearing loss, hypertension, smoking, obesity, depression, physical inactivity, diabetes, excess alcohol consumption, traumatic brain injury, air pollution, and social isolation).^45^ Our study has added to new evidence by uncovering a critical, previously understudied detail: a significant sex-specific difference in this relationship between blood pressure metrics and dementia. Specifically, our findings demonstrate that women with the APOE ε4 allele and elevated blood pressure metrics have a much higher risk of dementia compared to their male counterparts with the same risk factors. This finding is particularly valuable as it was observed in a large, community-based cohort, which enhances its generalizability. By explicitly evaluating and highlighting this sex-specific disparity, our study moves beyond simply confirming an existing association and provides a more nuanced understanding of how these risk factors are associated with dementia. This provides new avenues for targeted risk assessment and prevention strategies, particularly for high-risk female populations.

In the study, we observed that while the absolute dementia risk for APOE ε4 carriers is similar across sexes compared to the specific baseline of non-carrier men (Table 4), there were significantly joint effects of the APOE ε4 and blood pressure metrics on dementia risk in exposed group compare to the nonexposed exposed in women and men (Table 5), resulting in an excess risk of 23% in the joint effect of APOE ε4 and ankle pressure to 46% in the joint effect of APOE ε4 and pulse pressure. An increased pulse pressure and ankle pressure often reflect a higher degree of arterial stiffness in the legs and is a marker of systemic atherosclerosis, including in the cerebral arteries. Elevated pulse pressure and ankle pressure may exacerbate this vulnerability by reducing cerebral blood flow, impairing the clearance of waste products, such as amyloid-beta, and increasing the risk of micro-hemorrhages.^46^^,^^47^ Neuroimaging studies show that high pulse pressure and/or ankle pressure is associated with greater white matter hyperintensity burden, hippocampal atrophy, and accelerated amyloid deposition.^46^, ^47^, ^48^, ^49^ Significant multiplicative interaction effects of sex with APOE ε4, and APOE ε4 with pulse pressure in women were observed, but not in men. These findings suggest a complex biological relationship where the magnitudes of the joint risk effect vary by sex. Further research is warranted to explore the potential for interaction effects with larger studies or pooled datasets.

The mechanisms underlying the stronger impact of vascular factors on dementia risk in women are multifactorial and remain incompletely understood. Several biological pathways are likely to contribute. First, hormonal transitions around menopause, particularly the decline in estrogen, have been linked to increased arterial stiffness, reduced cerebral blood flow, and heightened neuroinflammatory responses.^50^^,^^51^ Estrogen is believed to confer neuroprotection through antioxidant, anti-inflammatory, and vasodilatory effects; its loss may increase women's susceptibility to both vascular injury and APOE ε4-related neurodegeneration. Our findings align with a significant sex disparity in dementia risk: women aged ≥55 had a significantly higher dementia incidence than men in the same age group. Moreover, the detrimental effect of the APOE ε4 allele was more pronounced, yielding a greater relative hazard ratio for dementia compared to men. Second, social and behavioral factors may play a role. In our study, lower educational attainment was associated with poorer cognitive performance in both sexes, but women had a disproportionately higher representation in the lower education strata. Furthermore, women's longer life expectancy may expose them to prolonged vascular and neurodegenerative stress, whereas men may be more likely to experience fatal cardiovascular events earlier in life, introducing a competing risk that could attenuate observed incidence of dementia. Third, structural and functional differences in brain anatomy may contribute to sex disparities in dementia pathogenesis.^52^ Women have been shown to exhibit greater hippocampal atrophy in response to vascular insults, even when risk factor levels are similar.^53^ In addition, women may have less robust collateral circulation in critical brain regions such as the precuneus and posterior cingulate—areas with high metabolic demand and rich vascularization, rendering them more susceptible to ischemic damage.^54^ While our study benefits from a large, diverse cohort and long-term follow-up, it is limited by the lack of detailed pathophysiological and neuroimaging data. Future research should incorporate molecular markers, neuroimaging, and mechanistic assessments to elucidate causal pathways linking vascular risk, sex, APOE ε4, and cognitive outcomes.

Our study has several notable strengths that enhance the robustness and reliability of its findings. First, we utilized data from the ARIC study, one of the largest and longest-running community-based cohort studies in the United States. This provided a wealth of data for robust longitudinal analyses of vascular factors and cognitive outcomes over nearly three decades. Second, the study's commitment to methodological rigor is a key strength. The use of standardized protocols for measuring blood pressure, along with comprehensive cognitive assessments and adjudicated dementia diagnoses, ensures high levels of reliability and validity in our data. Third, the inclusion of APOE genotyping allowed us to evaluate genetic susceptibility and investigate its interaction with vascular risk factors in predicting dementia. This enabled us to explore the interplay between genetic and environmental risk factors. Finally, our data analysis approaches were specifically tailored to the research questions. We utilize age as the time scale in our Cox proportional hazards regression models. This robust approach is preferred in aging research because it effectively minimizes age-related confounding and bias. Given that dementia is strongly associated with aging, this method provides a more precise estimate of the association between risk factors and dementia risk. It should be noted that despite these strengths, several limitations of our study should be considered. First, the ARIC cohort is restricted to African American and White participants, which limits the generalizability of our findings to other racial and ethnic groups, such as Hispanic, Asian, and Native American populations. Second, because baseline data collection began in midlife, the study does not capture early-life exposures or developmental risk factors that may critically influence later-life cognitive outcomes. Third, as an observational study, we cannot completely exclude the potential for residual confounding, selection bias, and information bias, including regarding self-reported behavioral and health data. Nonetheless, the large sample size, rigorous study design, standardized data collection procedures and measurements, and advanced statistical methods employed in our analyses help mitigate these limitations and provide strong evidence on vascular and genetic contributions to cognitive aging.

In conclusion, our study extends prior research as one of the first to explicitly quantify sex differences in the associations of vascular factors with cognitive decline and dementia risk. We demonstrate that these associations are significantly stronger in women than in men. Furthermore, we identified a significant joint effect of vascular factors and APOE ε4 allele on dementia risk, with women exhibiting a disproportionately higher relative excess risk from this combination. These critical results underscore the importance of incorporating sex-specific perspectives into vascular risk assessment, management, and global dementia prevention strategies.

## Contributors

LL conceived and designed the study, led the data analysis, interpreted the findings, drafted and made the decision to submit the report for publication. JH and SC participated in the data analysis. XZ, ZL, JCL, NS, JJL, RDG, and HJE critically reviewed and revised the report. All authors discussed the analytical approaches and results and contributed to the final version of the report.

## Data sharing statement

Data from the ARIC study can be accessed, with appropriate approvals, through the National Heart, Lung, and Blood Institute's Biospecimen and Data Repository Information Coordinating Center or by contacting the ARIC Coordinating Center.

## Declaration of interests

None.
