# Supplementary material for: Sex differences in the association of vascular risk and APOE Genotype with cognitive decline and dementia: evidence from a U.S. longitudinal study
Source: Lancet Reg Health Am. 2025 Dec 26;54:101346. doi: 10.1016/j.lana.2025.101346 (PMC12796570; doi:10.1016/j.lana.2025.101346)
Supplement: Supplementary Tables [file mmc1.pdf]

Sex Differences in the Association of Vascular Risk and APOE Genotype on Cognitive Decline and Dementia: Evidence from a U.S. Longitudinal Study

Longjian Liu[a], Jintong Hou[a], Saishi Cui[a], Xiaopeng Zhao[b], Zuolu Liu[c], J. Craig Longenecker[a], Nathalie S. May[d], Jin Jun Luo[e], Rose Ann DiMaria-Ghalili[f], and Howard J. Eisen[g]

Supplementary Tables

Supplementary Tables - L Liu et al.

Supplementary Table 1 Baseline characteristics of participants by dementia status during the follow-up **(N=12,268)**

	**Women (n=6,751)**					**Men (n=5,517)**				
	Non-dementia (n=5,152)		dementia (n=1,599)			Non-dementia (n=4,418)		dementia (n=1,099)		
	Mean, no	(±SD),(%)	Mean, no	(±SD),(%)	p-value	Mean, no	(±SD),(%)	Mean, no	(±SD),(%)	p-value
**Continuous Var, mean**, (SD)										
Age, year	53.1	(5.5)	56.2	(5.3)	**<0.0001**	54.1	(5.7)	56.1	(5.4)	**<0.0001**
SBP, mm Hg	118.8	(19.0)	121.9	(18.6)	**<0.0001**	121.3	(17.2)	122.4	(16.3)	**0.036**
DBP, mm Hg	71.7	(10.8)	72.6	(10.4)	**0.0035**	74.9	(10.9)	75.5	(10.8)	0.12
Ankle BP, mm Hg	138.4	(23.9)	144.0	(24.4)	**<0.0001**	152.0	(22.8)	155.0	(23.0)	**0.0001**
Brachial BP mm Hg	124.2	(20.9)	127.7	(21.2)	**<0.0001**	130.1	(18.4)	131.8	(17.8)	**0.0057**
Ankle to brachial index	1.11	(0.13)	1.12	(0.14)	**0.0030**	1.16	(0.14)	1.16	(0.14)	0.46
BMI, kg/m^2	27.5	(6.0)	28.1	(6.0)	**0.0008**	27.5	(4.1)	27.6	(3.9)	0.40
Log-CRP, mg/L	1.44	(0.80)	1.47	(0.83)	0.28	1.26	(0.72)	1.17	(0.67)	**<0.0001**
Cystatin C, mg/L	0.87	(0.34)	0.87	(0.19)	0.30	0.92	(0.26)	0.90	(0.18)	**0.0030**
**Categorical var, no. (%)**										
African American	1280	(24.8)	491	(30.7)	**<0.0001**	791	(17.9)	244	(22.2)	**0.0011**
Whites	3872	(75.2)	1108	(69.3)		3627	(82.1)	855	(77.8)	
Education, %					**<0.0001**					**<0.0001**
<High school	986	(19.2)	436	(27.3)		857	(19.4)	289	(26.3)	
High school	1985	(38.6)	596	(37.3)		1218	(27.6)	293	(26.7)	
>High school	2174	(42.3)	566	(35.4)		2336	(54.7)	516	(47.0)	
Smoking status					**<0.0001**					**<0.0001**
Never	2657	(51.6)	931	(58.2)		1256	(28.4)	349	(31.8)	**<0.0001**
Former	1220	(23.7)	351	(22.0)		1953	(44.2)	535	(48.7)	
Current	1268	(24.7)	317	(19.8)		1208	(27.4)	215	(19.6)	
Chronic conditions, no, %										
Hypertension	1719	(33.5)	665	(41.9)	**<0.001**	1499	(34.2)	391	(36.0)	0.26
Diabetes	531	(10.4)	185	(11.7)	0.15	485	(11.0)	126	(11.5)	0.67
CHD	142	(2.8)	33	(2.1)	0.13	405	(9.2)	69	(6.3)	**0.0020**
Heart failure	294	(5.8)	81	(5.1)	0.33	117	(2.7)	29	(2.7)	0.97
Stroke	283	(5.5)	100	(6.3)	0.25	214	(4.8)	56	(5.1)	0.73
APOE ε4 genotype, %					**<0.0001**					**<0.0001**
ε4 copy no. = 0	3752	(72.8)	936	(58.5)		3180	(72.0)	639	(58.1)	
ε4 copy no. = 1	1309	(25.4)	574	(35.9)		1161	(26.3)	407	(37.0)	
ε4 copy no. = 2	91	(1.8)	89	(5.6)		77	(1.7)	53	(4.8)	

BP: Blood pressure. SBP: Systolic blood pressure. DBP: Diastolic BP. BMI: Body mass index. Log-CRP: Log value of C-Reactive Protein. CHD: Coronary heart disease. Missing data were excluded in the baseline description.

Supplementary Table 2. Association of SBP, ankle and brachial pressure with risk of cognitive decline by sex (N=9,226)

	**Women**						**Men**					
BP associated with	**Model 1**			**Model 2**			**Model 1**			**Model 2**		
cognitive decline	β	(SE)	p-value	β	(SE)	p-value	β	(SE)	p-value	β	(SE)	p-value
SBP per 10 mm Hg												
Global ZS	-0.033	(0.005)	**<0.0001**	-0.013	(0.005)	**0.006**	-0.013	(0.007)	0.06	-0.006	(0.006)	0.36
DSST_ZS	-0.034	(0.006)	**<0.0001**	-0.012	(0.006)	**0.037**	-0.006	(0.008)	0.40	0.002	(0.007)	0.80
DWRT_ZS	-0.022	(0.007)	**0.0017**	-0.013	(0.007)	0.07	-0.016	(0.009)	0.08	-0.011	(0.009)	0.26
WFT_ZS	-0.044	(0.007)	**<0.0001**	-0.014	(0.007)	**0.049**	-0.016	(0.010)	0.11	-0.008	(0.009)	0.40
PP per 10 mm Hg												
Global ZS	-0.059	(0.007)	**<0.0001**	-0.034	(0.006)	**<0.0001**	-0.031	(0.009)	**0.001**	-0.013	(0.009)	0.12
DSST_ZS	-0.054	(0.008)	**<0.0001**	-0.026	(0.008)	**0.0011**	-0.041	(0.011)	**<0.001**	-0.018	(0.010)	0.07
DWRT_ZS	-0.048	(0.010)	**<0.0001**	-0.036	(0.010)	**<0.1001**	-0.025	(0.013)	0.06	-0.013	(0.013)	0.34
WFT_ZS	-0.074	(0.010)	**<0.0001**	-0.039	(0.010)	**<0.1001**	-0.028	(0.014)	**0.040**	-0.010	(0.013)	0.45
Ankle BP per 10 mm Hg												
Global ZS	-0.026	(0.004)	**<0.0001**	-0.013	(0.004)	**0.0008**	-0.009	(0.005)	0.08	-0.010	(0.004)	**0.021**
DSST_ZS	-0.026	(0.005)	**<0.0001**	-0.012	(0.005)	**0.0097**	0.000	(0.005)	0.99	-0.004	(0.005)	0.41
DWRT_ZS	-0.013	(0.006)	**0.022**	-0.007	(0.006)	0.22	-0.009	(0.007)	0.21	-0.009	(0.007)	0.19
WFT_ZS	-0.040	(0.006)	**<0.0001**	-0.019	(0.006)	**0.0008**	-0.017	(0.007)	**0.0151**	-0.018	(0.007)	**0.0079**
Brachial BP per 10 mm Hg												
Global ZS	-0.039	(0.004)	**<0.0001**	-0.017	(0.004)	**<0.0001**	-0.019	(0.006)	**0.0021**	-0.008	(0.006)	0.17
DSST_ZS	-0.039	(0.005)	**<0.0001**	-0.016	(0.005)	**0.0031**	-0.011	(0.007)	0.13	0.002	(0.006)	0.70
DWRT_ZS	-0.024	(0.006)	**<0.0001**	-0.014	(0.007)	**0.042**	-0.023	(0.009)	**0.0064**	-0.016	(0.009)	0.07
WFT_ZS	-0.053	(0.007)	**<0.0001**	-0.022	(0.007)	**0.0008**	-0.022	(0.009)	**0.0127**	-0.010	(0.008)	0.25
ABI in quadratic												
Global ZS	-0.260	(0.293)	0.38	-0.064	(0.274)	0.82	-0.653	0.310	**0.035**	-0.487	(0.281)	0.08
DSST_ZS	-0.740	(0.358)	**0.039**	-0.502	(0.338)	0.14	-0.681	(0.352)	0.05	-0.399	(0.316)	0.21
DWRT_ZS	-0.322	(0.420)	0.44	-0.166	(0.419)	0.69	-0.563	(0.438)	0.20	-0.461	(0.433)	0.29
WFT_ZS	0.282	(0.431)	0.51	-0.064	(0.274)	0.82	-0.716	(0.453)	0.11	-0.487	(0.281)	0.08

BP: Blood pressure. SBP: Systolic BP. PP: Pulse pressure. ABI: Ankle - brachial index. Global ZS: Mean of DSST_ZS, DWRT_ZS, and WFT_ZS. DSST_ZS: Z score of digit symbol substitution test.

DWRT_ZS: Z score of delayed word recall test. WFT_ZS: Z score of word fluency test. Model 1. Adjusted for age and race/ethnicity.

Model 2: Adjusted for age, race/ethnicity, education, body mass index (BMI), smoking, Cystatin C, CRP, and APOE gene.

Supplementary Table 3. Hazards ratio (95%CI) of dementia associated with vascular factors and APOE ε4 by sex (N=9,226)

	Women						Men					
	Model 1			Model 2			Model 1			Model 2		
	HR	(95%CI)	p-value	HR	(95%CI)	p-value	HR	(95%CI)	p-value	HR	(95%CI)	p-value
SBP/DBP mm Hg												
<120 / <80	1			1			1			1		
120-129 / <80	1.11	(0.90-1.36)	0.34	1.09	(0.89-1.34)	0.393	0.99	(0.77-1.26)	0.91	0.96	(0.75-1.23)	0.75
130-139 / 80-89	1.12	(0.91-1.37)	0.29	1.12	(0.91-1.37)	0.284	1.03	(0.82-1.28)	0.82	1.05	(0.84-1.32)	0.64
≥140 / ≥ 90	1.28	(1.12-1.46)	**0.0002**	1.25	(1.09-1.43)	**0.0017**	1.11	(0.94-1.30)	0.23	1.07	(0.90-1.27)	0.44
Elevated SBP mm Hg												
No	1			1			1			1		
Yes	1.04	(0.91-1.20)	0.55	1.04	(0.90-1.19)	0.61	0.91	(0.76-1.09)	0.30	0.90	(0.75-1.07)	0.24
Elevated PP, mm Hg												
No	1			1			1			1		
Yes	1.10	(0.96-1.25)	0.16	1.09	(0.95-1.24)	0.22	0.96	(0.80-1.14)	0.61	0.95	(0.80-1.14)	0.61
Elevated Ankle BP mm Hg												
No	1			1			1			1		
Yes	1.09	(0.94-1.26)	0.27	1.04	(0.89-1.21)	0.61	0.96	(0.83-1.12)	0.62	0.95	(0.82-1.11)	0.54
Elevated Brachial BP mm Hg												
No	1			1			1			1		
Yes	1.30	(1.13-1.49)	**0.0002**	1.21	(1.05-1.39)	**0.0072**	1.24	(1.05-1.45)	**0.011**	1.20	(1.02-1.42)	**0.030**
ABI in group												
Decreased	0.95	(0.81-1.11)	0.50	0.90	(0.77-1.05)	0.19	1.63	(1.27-2.09)	**<0.0001**	1.63	(1.27-2.09)	**<0.0001**
Normal	1			1			1			1		
Elevated	1.00	(0.82-1.22)	0.99	1.00	(0.82-1.22)	1.00	0.91	(0.74-1.12)	0.37	0.95	(0.77-1.17)	0.62
APOE ε4 genotype, %												
ε4 copy no.=0	1			1			1			1		
ε4 copy no.=1	1.79	(1.59-2.02)	**<0.0001**	1.80	(1.59-2.03)	**<0.0001**	1.72	(1.48-1.99)	**<0.0001**	1.72	(1.49-2.00)	**<0.0001**
ε4 copy no.=2	4.25	(3.30-5.47)	**<0.0001**	4.28	(3.32-5.51)	**<0.0001**	2.77	(1.97-3.88)	**<0.0001**	2.83	(2.02-3.97)	**<0.0001**

HR: Hazards ratio. BP: Blood pressure. SBP/DBP: Systolic / diastolic blood pressure. PP: Pulse pressure. ABI: Ankle - brachial index. APOE: Apolipoprotein E. Hazard ratios of dementia associated with blood pressure metrics were estimated by using baseline age plus follow-up year as time variable for more robustly controlling age effect on the study outcomes.

Model 1. Adjusted race/ethnicity and education. Model 2: Adjusted race/ethnicity, education, BMI, smoking. Cystatin C, CRP, and APOE gene.

Model 2: Adjusted for race/ethnicity, education, body mass index, smoking, Cystatin C, CRP, and APOE gene. In Model 2 when examining association between APOE e4 and risk of dementia, APOE gene was not adjusted because it was exposure.
